# *Mycoplasma genitalium* Endocarditis in Prosthetic Aortic Valve 

**DOI:** 10.3201/eid2910.221639

**Published:** 2023-10

**Authors:** Gokul Ramakrishnan, Iona Kronig, Nadia Gaïa, Vladimir Lazarevic, Jacques Schrenzel

**Affiliations:** Geneva University Hospitals, Geneva, Switzerland (G. Ramakrishnan, I. Kronig, J. Schrenzel);; University of Geneva, Geneva (N. Gaïa, V. Lazarevic, J. Schrenzel)

**Keywords:** *Mycoplasma genitalium*, bacteria, infective endocarditis, prosthetic heart valve, broad-range 16S rRNA PCR, Switzerland

## Abstract

We report a case of *Mycoplasma genitalium* endocarditis in a prosthetic heart valve of a woman who sought care in Switzerland for acute aortic valve dysfunction 3 years after valve replacement. This unusual manifestation of infection with this bacterium was diagnosed using broad-range PCR despite suspicion of a mechanical disinsertion.

Infective endocarditis is a life-threatening infection of the cardiac endothelium that can manifest as a new cardiac murmur, heart failure, valve vegetations, or sepsis. The diagnosis is based on a combination of clinical, echocardiographic, and microbiological features as outlined by the modified Duke criteria (*1*). Identifying the causative pathogen, along with clinical factors such as the nature of the infected valve (native or prosthetic) and epidemiology (community-acquired or hospital-acquired), is fundamental in defining the optimal therapy. Most pathogens that cause these infections, including *Staphylococcus*, *Streptococcus*, *Enterococcus*, *Haemophilus* spp., *Aggregatibacter actinomycetemcomitans*, *Cardiobacterium hominis*, *Eikenella corrodens*, and *Kingella kingae*, are identified by positive blood cultures. However, 2.5%–31% of cases (*2*) remain blood culture–negative because of prior antimicrobial treatment, intracellular organisms, or fastidious organisms. New organisms (e.g., *Malassezia* spp.) that warrant specific therapies have been recently reported (*3*). In those cases, serologic testing, excised valve microbiology, autoimmunohistochemistry (*4*), and molecular tools such as broad-range (16S rDNA) PCR and next-generation sequencing (NGS) may be necessary.

*Mycoplasma genitalium* is a very small, fastidious, and slow-growing pathogen. It is sexually transmitted and found primarily in the lower genital tract, capable of causing chronic infections. *M. genitalium* lacks a cell wall, making β-lactam, fosfomycin, and glycopeptide antimicrobials ineffective (*5*).

The main clinical manifestations of *M. genitalium* include infections at various locations in the genital tract in male and female patients. However, the direct evidence of *M. genitalium* pathogenicity is weak and often difficult to ascertain because of concomitant sexually transmitted pathogens. Furthermore, up to 94.4% male and 56.2% female case-patients may be asymptomatic carriers (*6*). We report an unusual case of *M. genitalium* endocarditis in a prosthetic heart valve of a woman who sought care in Switzerland.

## The Study

A 42-year-old woman from Portugal visiting her partner in Switzerland sought care for viral pneumonia in March 2022. Her medical history included systemic lupus erythematosus diagnosed in 2000 (on azathioprine), a mechanical aortic valve placed in 2019 (St. Jude Medical, https://www.cardiovascular.abbott) (on acenocoumarol), and a resection of uterine polyps in November 2021. She was initially hospitalized for 5 days; symptoms were fever and rhinovirus-positive nasal swab PCR, negative blood cultures, a C-reactive protein (CRP) of 142 mg/L, and mild heart failure (N-terminal prohormone of brain natriuretic peptide of 2,100 ng/L). Results of a thoracic computed tomography (CT) scan were compatible with pneumonia. She reported a severe penicillin allergy and exposure to cats. Treatment was 5 days of levofloxacin for a suspected bacterial superinfection and oral diuretics; she was discharged after satisfactory clinical improvement.

Ten days later, the woman again sought care for dyspnea, orthopnea, and dry cough. She was subfebrile (37.8°C) and had fine crackles in both lung bases; she required 2 L of nasal oxygen. Her blood tests showed a CRP of 20 mg/L and NT-proBNP of 3,200 ng/L; she was admitted for intravenous diuretics. Transthoracic echocardiography, transesophageal echocardiography, and a cardiac CT scan revealed severe prosthetic valve regurgitation with a posterior dehiscence (≈1/3 of the circumference) associated with a rocking motion of the prosthetic valve suggesting disinsertion, without vegetations (Figure). In a multidisciplinary meeting, hospital staff agreed on a likely diagnosis of prosthetic valve endocarditis; she was prescribed a treatment of ciprofloxacin and vancomycin.

**Figure:**
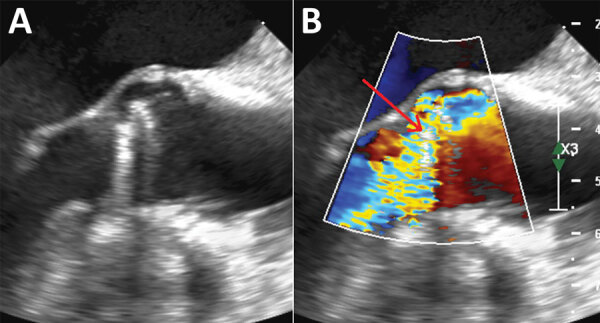
Trans-esophageal echocardiography without doppler (A) and with doppler (B) of the mechanical aortic heart valve in a woman diagnosed with *Mycoplasma genitalium* endocarditis. Red arrow indicates severe paraprosthetic regurgitation.

The next day, the patient underwent aortic valve replacement with a Bentall procedure. The surgeons strongly suspected that the initial valve implantation had not accounted for valve size or angle of implantation. There was no macroscopic evidence of endocarditis; prolonged blood cultures, valve cultures, aortic biopsy cultures and serologic tests for *Legionella pneumophila*, *Chlamydia pneumoniae*, *Brucella* spp., *Bartonella* spp., and *Coxiella burnetii* were all negative. No *Bartonella* PCR was performed on the patient’s blood or on the cardiac material. Nevertheless, the broad-range (16S rDNA; *Escherichia coli* positions 10–806) PCR and sequencing (*7*) performed on excised heart valve material identified *M. genitalium* with 100% match.

From the heart valve homogenate suspension in brain heart infusion broth, we extracted DNA using the bacterial DNA enrichment procedure including the Liberase-pretreatment (*8*) and spiking with 1,000 cells of each *Allobacillus halotolerans* and *Imtechella halotolerans* (ZymoBIOMICS Spike-in control I; Zymo Research, https://www.zymoresearch.com) before the bacterial lysis step. After 2 × 151 paired-end sequencing (Nextera DNA Flex/iSeq 100; Illumina, https://www.illumina.com), which generated 1,432,610 raw read pairs, we performed read quality check and removal of duplicated and low-complexity reads (*8*). From the resulting dataset (801,183 reads), we removed human-derived reads (n = 789,325) and then classified nonhuman reads (n = 11,858; European Nucleotide Archive accession no. PRJEB60931) against a custom database (*8*) using Kraken 2 (*9*) and Bracken (*10*). Classified reads mostly corresponded to *M. genitalium* (n = 11,113), followed by *Cutibacterium acnes*, a common reagent contaminant (n = 60), and *A. halotolerans* (spike organism) (n = 40). We assembled reads assigned to *M. genitalium* (*11*) into 366 contigs, with a sequencing depth of 5.6´, a predicted genome coverage of 84.6% (*12*) and an average nucleotide identity (*13*) of 99.78% to the *M. genitalium* type strain G37. The analysis of the genome assembly revealed the A2059G mutation (by *E*. *coli* numbering) in the 23S rRNA gene, known to confer macrolide resistance (*14*), and no mutations associated with quinolone resistance in *gyrA* and *parC*.

On day 5 after surgery, we initiated a treatment of levofloxacin (500 mg 2×/d) for 2 weeks, followed by moxifloxacin (400 mg 1×/d) with doxycycline (100 mg 2×/d). This treatment plan was largely motivated by the increasing prevalence of macrolide resistance worldwide (51% in 2016–2017) and the relatively less common fluoroquinolone resistance in Europe (*15*).

In her follow-up consultation after 4 weeks of treatment, the patient had a diffuse rash and arthralgia. Suspecting possible drug-related rash, we stopped doxycycline but continued moxifloxacin. A skin biopsy and immunology consultation revealed a flare of lupus erythematosus, which was managed with oral prednisone (20 mg/d). After 8 weeks of treatment, her immunologic symptoms improved, the cardiac echocardiography was unchanged, and moxifloxacin was stopped. At 9 months, she was completely asymptomatic.

## Conclusions 

We report a case of *M. genitalium* endocarditis that we identified and managed through molecular diagnostic tools in a situation in which valvular disinsertion was macroscopically suspected. We are not aware of previously documented cases of *M. genitalium* endocarditis, although 13 cases of *M. hominis*, 4 cases of *M. pneumoniae,* and 1 case of *M. salivarium* endocarditis have been reported (Appendix Table). *M. hominis* endocarditis has generally been identified in patients with heart conditions or prosthetic valves and has led to valve dehiscence and abscesses, prompting surgery in 2 cases that resulted in heart transplantation. *M. pneumoniae* endocarditis appears to affect native heart valves; 2 of 4 cases required valve replacement. Both species were principally diagnosed using broad-range PCR and were generally treated with a combination of macrolide or fluoroquinolone with doxycycline for 2–8 weeks, with overall good outcomes.

The diagnosis in this case was complicated by an unusual manifestation, the fastidious nature of the bacterium, recent antimicrobial drug exposure, and intraoperative structural findings consistent with a valve disinsertion. Our hypothesis of a transient bacteremia after a recent gynecologic procedure and subsequent invasion of a prosthetic heart valve could explain our patient’s pathogenesis. A lack of detailed information regarding that recent procedure was a limiting factor. Indeed, the value of performing broad-range PCR on cardiac valve samples cannot be understated. Furthermore, through NGS we confirmed the result, excluded the presence of other pathogens, and predicted antimicrobial resistance. Therefore, we recommend the systematic use of molecular assessment such as broad-range PCR or NGS in similar cases.

**Appendix.** Additional information about cases of *Mycoplasma* spp. endocarditis.
