# Supplementary material for: Mycoplasma genitalium Endocarditis in Prosthetic Aortic Valve
Source: Emerg Infect Dis. 2023 Oct;29(10):2164–6. doi: 10.3201/eid2910.221639 (PMC10521615; doi:10.3201/eid2910.221639)
Supplement: Supplementary file 1 — Appendix. Additional information about cases of Mycoplasma spp. endocarditis. [file 22-1639-Techapp-s1.pdf]

Article DOI: http://doi.org/10.3201/eid2910.221639

EID cannot ensure accessibility for supplementary materials supplied by authors. Readers who have difficulty accessing supplementary content should contact the authors for assistance.

Mycoplasma genitalium Endocarditis in Prosthetic Aortic Valve

Appendix

Appendix Table. Summary of cases of *Mycoplasma spp.* endocarditis reported in the literature*

Patient	Cardiac illness	Risk factors	Presentation	Echocardiography	Diagnosis	Valve replaced?	Antibiotic treatments	Follow-up
M. pneumoniae								
M21 (*1*)	Pericarditis	Long term penicillin V	Weight loss, fever	Severe AS, suspicious mitral valve	Serology	No	Penicillin G + Gentamicin 6wk, then oxytetracycline 4wk	Treatment failure, AVR after 3mo
M15 (*2*)	None	None	Fever (2mo), pulmonary emboli	Right ventricular vegetation	Culture	Yes	Ceftriaxone + Clarithromycin 3wk, then levofloxacin 6mo	?
F50 (*3*)	None	None	Mitral valve insufficiency, post-viral	Mitral valve chordae rupture	Serology	Yes	Linezolid + Ceftriaxone + Gentamicin + Azithromycin 10d	NAD at 12mo
F29 (*4*)	None	None	Fever, myalgia, weight loss	Pulmonary valve	Serology	No	Doxycycline 2wk	NAD at 2wk
M. hominis								
F25 (*5*)	2xAVR, endocarditis	Lupus	Aortic insufficiency, fever, polyarthralgia, post-AVR	None	Culture	Yes	Clindamycin + Rifampin 6wk, then doxycycline 4wk	HT at 2mo
M46 (*6*)	MVR	None	Progressive dyspnea	Mitral valve chordae rupture	Culture	Yes	Vancomycin + Amikacin	RIP after 2nd MVR in less than 2wk
M33 (*7*)	Mitral valve repair	None	Fever, dyspnea	Mitral valve dehiscence	16S rDNA PCR	Yes	Doxycycline 4wk	NAD at 24mo
F4 (*8*)	VSD	↓IgG	Fevers post-VSD repair	N/A	Culture	No	Clindamycin + Levofloxacin	RIP
F48 (*9*)	AVR	None	Fever, dyspnea	AS, AVR dysfunction	Culture	Yes	Doxycycline + Levofloxacin 8wk	NAD at 12mo

Patient	Cardiac illness	Risk factors	Presentation	Echocardiography	Diagnosis	Valve replaced?	Antibiotic treatments	Follow-up
M40 (*10*)	AVR and MVR	None	Fever, icterus	MR, dehiscence	Culture, 16S rDNA PCR	Yes	Doxycycline + Clindamycin 8wk	NAD at 12mo
M57 (*11*)	AVR	None	Chest pain, dyspnea, syncope	AR, sub-valvular obstruction	16S rDNA PCR	Yes	Doxycycline IV, then Moxifloxacin	NAD at 3mo
M74 (*12*)	AVR and MVR	None	Heart failure, vertigo	AS, abscess	16S rDNA PCR	Yes	Clindamycin + Doxycycline 9wk	NAD at 4mo
M54 (*13*)	AVR	None	Fever, chest pain	AR	16S rDNA PCR	Yes	Doxycycline + Levofloxacin 8wk	NAD at 2mo
F? (*14*)	TVR	IVDU	Postpartum, fever, cough, DAMA	TS	16S rDNA PCR	Yes	Co-amoxicillin 8wk	RIP after 4mo
M28 (*15*)	AVR	HBV	5th episode of valve dehiscence	Abscess	16S rDNA PCR	Yes (HT)	Moxifloxacin 8wk	NAD at 24mo
M67 (*16*)	ICD	None	Chest pain, heart failure	Pericardial effusion only	Culture, PCR	No (no valvular disease)	Moxifloxacin + Doxycycline 4wk	NAD at 10mo
M70 (*17*)	AVR	Diabetes	Dyspnea, fever	AR	16S rDNA PCR	Yes	Doxycycline 8wk	NAD at 24mo
M. salivarium								
M69 (*18*)	None	Psoriasis, diabetes, NHL	Joint pains	Mitral valve vegetation	16S rDNA PCR	No	Moxifloxacin 6wk	NAD at 24–36mo

*AR, aortic regurgitation; AS, aortic stenosis; AVR, aortic valve replacement; CD, implantable cardioverter-defibrillator; DAMA, discharge against medical advice; HT, heart transplant; IVDU, IV drug user; mo, months; NAD, no appreciable disease; NHL, Non-Hodgkin lymphoma; MVR, mitral valve replacement; RIP, death; TS, tricuspid stenosis; TVR, tricuspid valve replacement; VSD, ventricular septal defect; wk, weeks.

References

1. Popat K, Barnardo D, Webb-Peploe M. *Mycoplasma pneumoniae* endocarditis. Br Heart J. 1980;44:111–2. PubMed https://doi.org/10.1136/hrt.44.1.111

2. Scapini JP, Flynn LP, Sciacaluga S, Morales L, Cadario ME. Confirmed *Mycoplasma pneumoniae* endocarditis. Emerg Infect Dis. 2008;14:1664–5. PubMed https://doi.org/10.3201/eid1410.080157

3. Augustatou C, Glynos C, Cokkinos P, Papaparaskevas J. Culture-negative endocarditis due to *Mycoplasma pneumoniae.* OA Case Reports. 2013 [cited 2023 Aug 29]. https://doi.org/10.13172/2052-0077-2-2-408.

4. Dawood H, Nasir S, Khair RM, Dawood M. Infective endocarditis secondary to *Mycoplasma pneumoniae*. Cureus. 2021;13:e17461. PubMedhttps://doi.org/10.7759/cureus.17461

5. Cohen JI, Sloss LJ, Kundsin R, Golightly L. Prosthetic valve endocarditis caused by *Mycoplasma hominis.* Am J Med. 1989;86:819–21. PubMed https://doi.org/10.1016/0002-9343(89)90479-8

6. Blasco M, Torres L, Marco ML, Moles B, Villuendas MC, García Moya JB. Prosthetic valve endocarditis caused by *Mycoplasma hominis.* Eur J Clin Microbiol Infect Dis. 2000;19:638–40. PubMed https://doi.org/10.1007/s100960000333

7. Fenollar F, Gauduchon V, Casalta J-P, Lepidi H, Vandenesch F, Raoult D. *Mycoplasma endocarditis*: two case reports and a review. Clin Infect Dis. 2004;38:e21–4. PubMed https://doi.org/10.1086/380839

8. Dominguez SR, Littlehorn C, Nyquist A-C. *Mycoplasma hominis* endocarditis in a child with a complex congenital heart defect. Pediatr Infect Dis J. 2006;25:851–2. PubMed https://doi.org/10.1097/01.inf.0000232639.81762.d4

9. Hidalgo-Tenorio C, Pasquau J, López-Checa S, López-Ruz MA. Endocarditis due to *Mycoplasma hominis*. Enferm Infecc Microbiol Clin. 2006;24:470–1. PubMed https://doi.org/10.1157/13091789

10. Jamil HA, Sandoe JAT, Gascoyne-Binzi D, Chalker VJ, Simms AD, Munsch CM, et al. Late-onset prosthetic valve endocarditis caused by *Mycoplasma hominis,* diagnosed using broad-range bacterial PCR. J Med Microbiol. 2012;61:300–1. PubMed https://doi.org/10.1099/jmm.0.030635-0

11. Hussain ST, Gordon SM, Tan CD, Smedira NG. *Mycoplasma hominis* prosthetic valve endocarditis: the value of molecular sequencing in cardiac surgery. J Thorac Cardiovasc Surg. 2013;146:e7–9. PubMed https://doi.org/10.1016/j.jtcvs.2013.03.039

12. Gagneux-Brunon A, Grattard F, Morel J, et al. (). Correction for Gagneux-Brunon et al., *Mycoplasma hominis,* a rare but true cause of infective endocarditis. J Clin Microbiol. 2016;54:244. https://doi.org/10.1128/JCM.02719-15.

13. Romeu Prieto JM, Lizcano Lizcano AM, López de Toro Martín Consuegra I, Largo Pau J, López Almodóvar LF, García Camacho E. Culture-negative endocarditis: *Mycoplasma hominis* infection. Rev Esp Cardiol (Engl Ed). 2015;68:1037–8. PubMed https://doi.org/10.1016/j.rec.2015.07.018

14. Kotaskova I, Nemec P, Vanerkova M, Malisova B, Tejkalova R, Orban M, et al. First report of *Sneathia sanguinegens* together with *Mycoplasma hominis* in postpartum prosthetic valve infective endocarditis: a case report. BMC Infect Dis. 2017;17:563. PubMed https://doi.org/10.1186/s12879-017-2654-8

15. Givone F, Peghin M, Vendramin I, et al. Salvage heart transplantation for *Mycoplasma hominis* prosthetic valve endocarditis: a case report and review of the literature. Transpl Infect Dis. 2020;22:e13249. https://doi.org/10.1111/tid.13249

16. Bustos-Merlo A, Rosales-Castillo A, Cobo F, Hidalgo-Tenorio C. Blood culture-negative infective endocarditis by *Mycoplasma hominis*: case report and literature review. J Clin Med. 2022;11:3841. PubMed https://doi.org/10.3390/jcm11133841

17. Kim MGJ, Payne S, Post J. A subacute presentation of *Mycoplasma hominis* prosthetic valve endocarditis. BMJ Case Rep. 2022;15:e252972. PubMed https://doi.org/10.1136/bcr-2022-252972

18. Scheen M, Attinger A, Huwyler T, Togni M, Puricel S. A mouth watering case of *Mycoplasma salivarium* native mitral valve endocarditis: case report. BMC Infect Dis. 2023;23:81. PubMed https://doi.org/10.1186/s12879-023-08048-8
